# Human Umbilical Cord Mesenchymal Stem Cells Encapsulated with Pluronic F-127 Enhance the Regeneration and Angiogenesis of Thin Endometrium in Rat via Local IL-1*β* Stimulation

**DOI:** 10.1155/2022/7819234

**Published:** 2022-06-18

**Authors:** Shuling Zhou, Yu Lei, Ping Wang, Jianying Chen, Liting Zeng, Ting Qu, Martin Maldonado, Jihua Huang, Tingting Han, Zina Wen, Erpo Tian, Xiangqian Meng, Ying Zhong, Jiang Gu

**Affiliations:** ^1^Jinxin Research Institute for Reproductive Medicine and Genetics, 66 Bisheng Road, Chengdu, 610066 Sichuan, China; ^2^Department of Pathology and Provincial Key Laboratory of Infectious Diseases and Immunopathology, Collaborative and Creative Center, Shantou University Medical College, 22 Xinling Road, Shantou, 515041 Guangdong, China; ^3^Department of Andrology, Chengdu Xi'nan Gynecological Hospital, 66 Bisheng Road, Chengdu, 610066 Sichuan, China; ^4^Department of Embryology, Chengdu Jinjiang Hospital for Maternal and Child Health Care, 3 San-guantang Road, Chengdu, 610066 Sichuan, China

## Abstract

Thin endometrium (< 7 mm) could cause low clinical pregnancy, reduced live birth, increased spontaneous abortion, and decreased birth weight. However, the treatments for thin endometrium have not been well developed. In this study, we aim to determine the role of Pluronic F-127 (PF-127) encapsulation of human umbilical cord mesenchymal stem cells (hUC-MSCs) in the regeneration of thin endometrium and its underlying mechanism. Thin endometrium rat model was created by infusion of 95% ethanol. Thin endometrium modeled rat uterus were treated with saline, hUC-MSCs, PF-127, or hUC-MSCs plus PF-127 separately. Regenerated rat uterus was measured for gene expression levels of angiogenesis factors and histological morphology. Angiogenesis capacity of interleukin-1 beta (IL-1*β*)-primed hUC-MSCs was monitored via quantitative polymerase chain reaction (q-PCR), Luminex assay, and tube formation assay. Decreased endometrium thickness and gland number and increased inflammatory factor IL-1*β* were achieved in the thin endometrium rat model. Embedding of hUC-MSCs with PF-127 could prolong the hUC-MSCs retaining, which could further enhance endometrium thickness and gland number in the thin endometrium rat model via increasing angiogenesis capacity. Conditional medium derived from IL-1*β*-primed hUC-MSCs increased the concentration of angiogenesis factors (basic fibroblast growth factor (bFGF), vascular endothelial growth factors (VEGF), and hepatocyte growth factor (HGF)). Improvement in the thickness, number of glands, and newly generated blood vessels could be achieved by uterus endometrium treatment with PF-127 and hUC-MSCs transplantation. Local IL-1*β* stimulation-primed hUC-MSCs promoted the release of angiogenesis factors and may play a vital role on thin endometrium regeneration.

## 1. Introduction

Thin endometrium is one of the main reasons for decreased clinical pregnancy, increased ectopic pregnancy rates, increased spontaneous abortion, and reduced live birth weight during application of assisted reproductive technology (ART) [1–3]. Thin endometrium is defined as endometrium thickness less than 7 mm [4]. The incidence of patients diagnosed as thin endometrium is from 2.4% to 8.5% [5, 6]. Endometrium thickness was strongly associated with success of fertility. Many treatments such as “hormonal,” “vascular,” and “growth factor” have been attempted to increase the endometrium thickness [7]. However, until now none of these has been proven effective. Thus, improving endometrium thickness to increase clinical pregnancy and live birth for infertile couples is still a challenge for the clinicians.

Mesenchymal stem cells (MSCs) are widely used in repairing damaged tissues and achieving regeneration by promoting local capillary angiogenesis, inhibiting fibrosis, and executing immunomodulatory functions [8]. MSCs can be isolated from various tissues such as adipose tissues, bone marrow, umbilical cord, and placenta. Human umbilical cord is one of most economic and easily collectable sources to isolate MSCs, which is usually the medical waste after delivery [9]. HUC-MSCs are characterized by their highly proliferative capability, low immunogenicity, and in particular angiogenesis, which are clinically utilized for tissue repairing, and treatments of degenerative and inflammatory diseases with intravenous administration or local incubation [10]. More than 90% of MSCs were retained in the liver, lungs, and spleen rather than other organs through intravenous administration [11]. Limited numbers and poorly survived MSCs would reside in the uterus with direct cell infusion into uterus cavity. Therefore, biodegradable scaffolds could be applied for retention of MSCs in the injury endometrium [12].

PF-127 is one of the United States Food and Drug Administration (USFDA)–approved thermosensitive biodegradable hydrogels for clinical application [13]. PF-127 exhibits as liquid solution phase at low temperature (4°C) and is able to transform into solid gel solution at body temperature [14]. PF-127 characterizes as porous structure, which could further enhance the therapeutic effects by extending half-lives of drugs in serum [15]. These characteristics enable PF-127 to load the extracellular vesicles or cells in low temperature and form gel in the irregularly damaged space to promote regeneration [16–19]. PF-127 transplanted with hUC-MSCs was applied to regenerate intrauterine adhesion (IUA) rat uterus [20]. However, the effect about encapsulation of PF-127 with hUC-MSCs to regenerate thin endometrium remains to be elucidated.

It is known that underlying mechanisms of hUC-MSCs-based regeneration therapy is associated with paracrine function [21]. Local microenvironment is one of the issues that could modulate paracrine activities of MSCs [22]. Exposure MSCs on local titanium (Ti) particle could dysregulate MSCs population and alter vessel formation, which further induce local chronic Inflammation [23]. Incubation of MSCs with tumor necrosis factor-alpha (TNF-*α*) would promote the secretion of proangiogenic cytokines, which induce angiogenesis and tissue repair [24]. Interferon-*γ* (IFN-*γ*)-treated MSCs was found to have enhanced prostaglandin E2 expression, which improves the antifibrotic ability of MSCs [25]. However, whether local microenvironment could enhance the expression of growth factors and angiogenesis factors of hUC-MSCs to regenerate thin endometrium is largely unknown. Moreover, the cellular and molecular mechanisms of regenerative effect on thin endometrium are yet to be discovered.

Thus, in the present study, we aim to investigate the therapeutic effects of PF-127 embedded hUC-MSCs on regeneration of thin endometrium. We found that PF-127-loaded hUC-MSCs could elevate angiogenesis with IL-1*β* stimulation, which further facilitated thin endometrium regeneration. Our findings may uncover a novel underlying mechanism for regeneration of thin endometrium.

## 2. Materials and Methods

### 2.1. Animals

Eight-week-old female Sprague-Dawley (SD) rats weighting 200-250 g were purchased from Chengdu Dossy Experimental Animals Co., Ltd. (Chengdu, Sichuan, China). After one-week adaption, the rats with consecutive 4-day estrous cycles were used in the experiment. All the animal experiments were carried out according to the guidelines indicated in the “Guide for the Care and Use of Laboratory Animals” [26]. The animal experiments were approved by the Ethics Review Board of Jinxin Research Institute for Reproductive Medicine and Genetics (approval ID: 2020YXLSD07).

### 2.2. Isolation and Culturing of hUC-MSCs

With the permission of the Ethics Review Board of Chengdu Jinjiang Hospital for Maternal and Child Health Care (approval ID: 2020SZLSD02), 3 fresh human umbilical cords were collected from the Chengdu Jinjiang Hospital for Maternal and Child Health Care. The consent forms were signed by the three donors. Approximately 10-cm human umbilical cord was received from full-term birth after Caesarean section. Umbilical cord was collected in sterilized laboratory bottle with Dulbecco's Phosphate-Buffered Saline (DPBS) (Thermo Fisher Scientific, Grand Island, New York, USA) containing 100 U/ml penicillin and 100 *μ*g/ml streptomycin (Thermo Fisher Scientific) on ice. Umbilical cord was cut into 3 cm pieces and rinsed with ice-cold DPBS to remove blood clots. Blood vessels were completely removed from umbilical cord by using scissors and forceps. Afterwards, umbilical cord was cut into small pieces (1 to 2 mm^3^) with scalpel. The small pieces of umbilical cord tissues were seeded into 10-cm dishes for 30-min incubation in 5% CO_2_ at 37°C without medium. Half hour later, 7 ml of complete growth medium consisting of minimum essential medium *α* (*α*-MEM) (Thermo Fisher Scientific) supplemented with 10% fetal bovine serum (FBS) (ExCell Bio, Taicang, Jiangsu, China) was added in the dishes to culture. After two weeks of culturing, small pieces of umbilical cord were removed. HUC-MSCs outgrowth from the explant was further passaged with TrypLE Express (Thermo Fisher Scientific) for characterization of cell transplantation.

### 2.3. Flow Cytometry

In order to perform flow cytometry analysis, the 3^rd^ to 5^th^ passage of 3 lines of hUC-MSCs were dissociated into single cells by TrypLE Express and followed by resuspended 1 × 10^5^ hUC-MSCs in 100 *μ*l of 1 mg/ml bovine serum albumin (BSA)/DPBS (Thermo Fisher Scientific). Cells were incubated with the following allophycocyanin (APC)-conjugated antibodies in dark for one hour, which included antihuman CD34, antihuman CD45, antihuman CD29, antihuman CD73, antihuman CD90, antihuman CD105, antihuman CD44, antihuman HLA-DR, and isotype controls (eBioscience, San Diego, California, USA). Cells were washed twice with ice cold 1 mg/ml BSA/DPBS and resuspended in 400 *μ*l of 1 mg/ml BSA/DPBS. Subsequently, these surface markers were evaluated by flow cytometer (BD Biosciences, San Jose, California, USA). Data was analyzed using FlowJo software (BD Biosciences).

### 2.4. Differentiation of hUC-MSCs

The multilineage differentiation potential of hUC-MSCs was evaluated by differentiation of 3^rd^ passage of hUC-MSCs into adipocytes, chondrocytes, and osteocytes. Adipogenic differentiation of hUC-MSCs was induced by 3 weeks of adipogenic differentiation medium culturing. Adipogenic differentiation medium was composed of Dulbecco's Modified Eagle Medium-low glucose (DMEM-LG) (Thermo Fisher Scientific) with 20% FBS (ExCell Bio) supplemented with 5 *μ*g/ml insulin, 0.1 *μ*M dexamethasone, 0.5 mM IBMX, and 60 *μ*M indomethacin (Sigma-Aldrich, St. Louis, Missouri, USA) [27]. Osteogenic differentiation of hUC-MSCSs was induced by osteogenic differentiation medium for 3 weeks. Osteogenic differentiation was performed in medium composed of Dulbecco's Modified Eagle Medium-high glucose (DMEM-HG) (Thermo Fisher Scientific) with 10% FBS (ExCell Bio) supplemented with 100 nM dexamethasone, 10 mM *β*-Glycerophosphate, 50 *μ*M ascorbic acid-2 phosphate, and 10 *μ*M vitamin D (Sigma-Aldrich). Chondrogenic differentiation was initiated by collection and centrifugation of hUC-MSCs in 15 ml of falcon tube. After centrifugation the medium was changed to chondrogenic differentiation medium. The medium was changed every second day. Chondrogenic differentiation medium was composed of DMEM-HG (Thermo Fisher Scientific) with 10% FBS (ExCell Bio) supplemented with 1× insulin, transferrin, and selenium (ITS-G) (Thermo Fisher Scientific), 100 *μ*g/ml of pyruvate (Gibco), 5.0 *μ*g/ml linoleic acid, 1.0 mg/ml bovine serum albumin, 100 nM dexamethasone, 50 *μ*g/ml L-ascorbic acid 2-phosphate, 40 *μ*g/ml of proline (Sigma-Aldrich), 100 ng/ml of bone morphogenetic protein-2 (BMP2) (PeproTech, Rocky Hill, New Jersey, USA), and 10 ng/ml of transforming growth factor *β*3 (TGF-*β*3) (PeproTech) [28]. Oil Red O staining was applied for detecting the formation of lipid droplets in the hUC-MSCs-derived adipocytes. Alizarin red staining was performed to visualize the calcium deposition in the hUC-MSCs-derived osteocytes. Alcian blue staining was used to verify the chondrocytes differentiation potential from hUC-MSCs. All the staining dyes were purchased from Sigma-Aldrich.

### 2.5. Preparation and Characterization of PF-127 and C/GB Hydrogel

PF-127 hydrogel was prepared with the following protocol as described previously [20]. PF-127 powder (Sigma-Aldrich) dissolved in *α*-MEM at a 20% concentration for overnight stirring at 4°C. PF-127 hydrogel was sterilized with 0.22-*μ*m filter (Millipore, Billerica, Massachusetts, USA). C/GP hydrogel was composed of chitosan solution and *β*-glycerophosphate solution [29–31]. The 2.2% chitosan solution was made up by dissolving 93% deacetylation chitosan powder (Haidebei, Dalian, Liaoning, China) in 0.1 M acetic acid. The 50% *β*-glycerophosphate solution was prepared in distilled water. Both 2.2% chitosan solution and 50% *β*-glycerophosphate solution were pass through 0.22-*μ*m filter (Millipore) and kept in 4°C for later using. The 50% *β*-glycerophosphate solution was added into 2.2% chitosan solution in the 5 : 1 volume ratio in an ice bath. To measure the gelation time of PF-127 and C/GP hydrogels, the prepared hydrogel was added into 50-ml falcon tube (BD Biosciences), which was placed into 37°C water bath. The tube was examined every 1 minute by inversion, until the hydrogel gelling.

### 2.6. Scanning Electron Microscope (SEM)

SEM was applied to observe the structure of PF-127 and C/GP. The samples were freeze-dried for 24 hours to obtain dehydrated hydrogels. Internal structure of dehydrated hydrogel was viewed by sputtering with gold-palladium for SEM imaging at 15 kV using a FEI Apreo SEM (Thermo Fisher Scientific). Porosity and pore size were measured with Fiji software [32–34].

### 2.7. Establishment of Thin Endometrium Rat Model

Eighteen female SD rats (200-250 g) with 4-day estrous cycles were used for modeling thin endometrium. All the rats were anesthetized with 4% of Nembutal (60 mg/kg). These rats were randomized into modeling group (*n* = 9) and control group (*n* = 9). Around 2-cm vertical incision was made to expose the right uterus. From bottom of right uterine horn, 1.5 cm of uterine horn was clipped with vascular clip. For the modeling group, 200 *μ*l of 95% ethanol was injected into right uterine horn for 3 minutes. Subsequently the uterine horn was washed twice with saline. For the control group, 200 *μ*l of saline was injected into right uterine horn for 3 minutes and washed with saline twice. Nine days after the thin endometrium modeling, rats were sacrificed. Modeled uterus and control uterus were collected for further quantitative q-PCR and hematoxylin and eosin (HE) staining.

### 2.8. Determination of Biocompatibility of PF-127 and C/GP Hydrogel in Rat

Eight female SD rats (200-250 g) were used to evaluate the biocompatibility of PF-127 and C/GP hydrogel in vivo. All the rats were anesthetized with 4% of Nembutal (60 mg/kg). Three hundred microliter of PF-127 and C/GP hydrogel was subcutaneously injected into forelimbs and hindlimbs on both sides. Four weeks later, all the rats were sacrificed. Tissues around the hydrogel injection site were collected for q-PCR.

### 2.9. Transplantation of PF-127-Encapsulated hUC-MSCs

Forty-eight rats were used to generate thin endometrium modeling by injection of 95% ethanol into the right side of uterine horns and rinsing twice with saline. To further investigate the efficiency of PF-127-encapsulated hUC-MSCs on reconstructing the function of destroyed endometrium, all the rats were randomly assigned into 4 groups. They consisted of a saline group (*n* = 12 uterine horns), PF-127 group (*n* = 12 uterine horns), hUC-MSCs group (*n* = 12 uterine horns), and PF-127-encapsulated hUC-MSCs group (*n* = 12 uterine horns). Nine days after thin endometrium modeling, 200 *μ*l of saline, PF-127 hydrogel, hUC-MSCs (5 × 10^6^), and PF-127 hydrogel-encapsulated hUC-MSCs (5 × 10^6^) were injected into the right side of the uterine horns for treatment. Nine days after the treatment, all the rats were sacrificed. The right side uterine horns were collected for q-PCR, TaqMan-based quantitative real-time PCR (TaqMan qPCR), HE, and immunohistochemistry (IHC) analyses.

### 2.10. Preparation of IL-1*β* Conditioned Medium

Three lines of hUC-MSCs were seeded into 6-well plate and grown until approximately 80%-90% confluency. Cells were switched into a-MEM medium for overnight starving. To generate the IL-1*β*-conditioned medium, cells were incubated in a-MEM medium with different concentrations of IL-1*β* (PeproTech) (0, 20, 40, and 100 ng/ml) for 24 hours. Subsequently, the cells cultured in various concentrations of IL-1*β* induction were collected for q-PCR. Condition medium was harvested for Luminex analysis and tube formation assay test.

### 2.11. HE Examination

Collected tissues were fixed in 4% paraformaldehyde (PFA) for 4 hours. All the fixed samples were automatically dehydrated with Leica TP1020 with standard protocol (Leica, Buffalo Grove, Illinois, USA). The dehydrated samples were embedded in paraffin and cut into 4-*μ*m sections (Leica). Afterwards, the sections were dewaxed and rehydrated in xylene and a graded series of ethanol. HE staining was applied to observe the structure of collected uterus. Fiji software was applied to evaluate the thickness of endometrium and gland number. The thickness of the uterine horn was determined by measuring the vertical distances from the lumen to the myometrium at 4 different directions (lateral and longitudinal side).

### 2.12. IHC Analysis

Sections were stained with the angiogenesis makers including antivascular endothelial growth factor (VEGFA) antibody (1 : 200, Abcam, Cambridge Biomedical Campus, Cambridge, UK) and anti-von Willebrand factor (VWF) antibody (1 : 200, Abcam). A number of endometrial glands in each section were manually counted under 4× magnification. The number of VEGFA and VWF-positive blood vessel density were counted from 5 randomly selected images of each section under 20× magnification.

### 2.13. q-PCR

Total RNA was extracted by RNAiso Plus (Takara, Dalian, Liaoning, China) following the manufacturer's protocol. One microgram of total RNA was transcribed to cDNA by using the PrimeScript RT reagent Kit with gDNA Eraser (Takara) in accordance with the manufacturer's instructions. Q-PCR reactions were performed using KAPA SYBR® FAST Universal Kit (Sigma-Aldrich) on a 7500 Real-Time PCR Systems (Thermo Fisher Scientific). The mRNA expression levels were normalized to *18S* and *B2M* in rat tissues and cultured cells, respectively. Data was analyzed based on 2^-*ΔΔ*Ct^ method [35].

The sequences of primers are summarized in Table 1:

### 2.14. TaqMan qPCR and DNA Calculation

Genomic DNA was extracted by GeneJET Genomic DNA Purification Kit (Thermo Fisher Scientific) following the manufacturer's protocol. Primers/probes were designed to detect human-specific DNA by targeting the a-satellite region of human chromosome 17 (Table 2) [36]. TaqMan qPCR reactions were performed using TaqMan™ Fast Advanced Master Mix (Thermo Fisher Scientific) on a 7500 Real-Time PCR Systems (Thermo Fisher Scientific). Five-fold serial dilutions of human DNA (5 ng, 1 ng, 0.2 ng, 0.04 ng, 0.008 ng, and 0.0016 ng) were applied to generate the standard curves. The amount of human DNA in hUC-MSCs and PF-127-encapsulated hUC-MSCs-treated uterus was calculated by established standard curves. To calculate the percentage of human DNA retained within the uterus, the amount human DNA identified in the treated uterus was divided by TaqMan qPCR input total DNA amount (40 ng).

### 2.15. Luminex Assay

The supernatant of conditioned medium was harvested and evaluated for angiogenesis markers with the Luminex immunobead platform following the manufacturer's protocol (Merck Millipore). Angiogenesis panels of bFGF, VEGF, and HGF were analyzed.

### 2.16. Tube Formation Assays

Tube formation assays were performed to evaluate the in vitro neovascularization capacity of conditioned medium with IL-1*β* induction [37]. Approximately 7.5 × 10^4^ human umbilical vein endothelial cells (HUVECs) were resuspended with 300 *μ*l conditioned medium from various concentration of IL-1*β* induction. Subsequently, the HUVECs were planted into 4-well plate coated with Matrigel (BD Biosciences, San Diego, California, USA). After 12-h incubation, the cells were fixed with 4% PFA for further analysis. Eight pictures per well were acquired from 3 independent cultures (20× magnification). Fiji software was employed to measure the tube length of HUVECs.

### 2.17. Statistical Analysis

Normal distribution of all the data was determined by using Shapiro–Wilk test. Brown-Forsythe was applied for equal variance test. If the data was normally distributed and had equal variance, Student's *t*-test was applied to test significance. If the data was normally distributed and had unequal variance, Welch's *t*-test was applied to test significance. If the data fail to pass normality test, Mann–Whitney rank sum test was used for significance test. *P* < 0.05 was considered statistically significant. Results were reported as the mean ± S.E.M (standard error of the mean). Data was analyzed using SigmaPlot software (SigmaPlot software, San Jose, California, USA). Figures were produced with GraphPad Prism 8 (GraphPad Prism 8, San Diego, California, USA).

## 3. Results

### 3.1. Culturing and Characterization of *hUC-MSCs*

**Figure 1:**
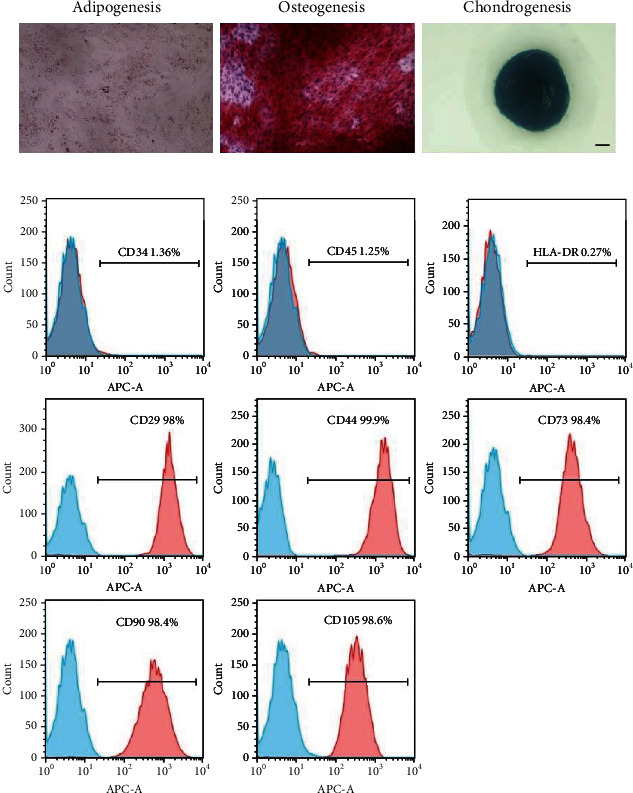
Characterization of hUC-MSCs. (a) Trilineage differentiation of hUC-MSCs. Adipocyte differentiation capacity of hUC-MSCs was confirmed by Oil Red O staining. Osteogenic differentiation potential of hUC-MSCs was verified by Alizarin red staining. Chondrogenic differentiation ability of hUC-MSCs was proved by Alcian blue staining. Scale bar = 1000 *μ*m. (b) Flow cytometry analysis of mesenchymal markers (CD34-, CD45-, HLA-DR-, CD29+, CD73+, CD90+, CD105+, and CD44+) and the corresponding isotype controls (mouse IgG1 Kappa, rat IgG2b Kappa, and mouse IgG2b Kappa).

To investigate the multipotency of hUC-MSCs, we performed the adipogenic, osteogenic, and chondrogenic differentiation assays. Three weeks after adipogenic differentiation, adipocytes could be detected with Oil Red O staining. Three weeks after osteogenic induction, 90% of hUC-MSCs differentiated into osteocytes, which were stained with Alizarin red. Three weeks after chondrogenic differentiation, chondrocytes could be observed by Alcian blue staining (Figure 1(a)). Flow cytometry was performed to characterize the typical MSC marker expression. HUC-MSCs were strongly positive for CD29 (98%), CD44 (99.8%), CD73 (98.4%), CD90 (98.4%), and CD105 (98.6%) but negative for CD34 (1.36%), CD45 (1.25%), and HLA-DR (0.27%) (Figure 1(b)). These results indicated that the isolated hUC-MSCs exhibited the typical MSCs markers.

### 3.2. Larger Porosity and Improved Biocompatible Was Observed in PF-127 Hydrogel Compared to C/GB Hydrogel

**Figure 2:**
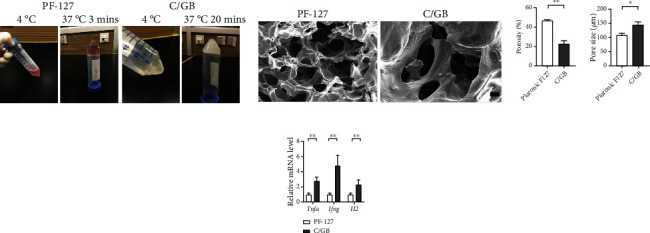
PF-127 hydrogel possesses larger interconnected pore and higher biocompatible capacity compared to C/GP hydrogel. (a) Photographs of PF-127 and C/GP hydrogel. (b) SEM images of PF-127 and C/GP after 24-hour freeze drying process. Scale bar = 300 *μ*m. (c) Quantification of porosity of PF-127 and C/GP hydrogel. Data was reported as mean ± S.E.M of 5 SEM images with 200× magnification, tested for significant difference (∗∗*P* < 0.01) using Student's *t*-test. (d) Comparison of pore size of PF-127 and C/GB hydrogel. Results were presented as mean ± S.E.M of approximately 50 pores from 5 SEM images with 100× magnification. Significant difference test was performed with Mann–Whitney rank sum test (∗*P* < 0.05). (e) Q-PCR analyzed the relative gene expression of inflammatory factors (*Tnfa*, *Ifng*, and *Il2*). Data was reported as mean ± S.E.M. Student's *t*-test was applied for significant difference (∗∗*P* < 0.01), *n* = 16.

Both PF-127 and C/GB hydrogel exhibited flowable liquid solution phase at 4°C. More swift gelling time at 37°C was achieved in PF-127 hydrogel (3 minutes) in comparison with C/GB hydrogel (20 minutes) (Figure 2(a)). Furthermore, SEM was performed to observe the structure of PF-127 and C/GB hydrogel. Porous structure could be observed in PF-127 and C/GB hydrogel (Figure 2(b)). Significantly larger porosity was observed in PF-127 hydrogel (47.04 ± 0.72%) compared to C/GB hydrogel (22.84 ± 3.15%) (*P* < 0.01) (Figure 2(c)). Wider pore size was found in C/GB hydrogel (145.28 ± 10.78 *μ*m) compared to PF-127 hydrogel (108.64 ± 5.84 *μ*m) (*P* < 0.05) (Figure 2(d)). Subsequently, PF-127 and C/GB hydrogel were subcutaneously administrated into rat forelimb and hindlimb to examine the in vivo biocompatible characteristics. Four weeks after transplantation, significantly lower gene expression level of *Tnfa*, *Ifng*, and *Il2* expressions were observed in PF-127 hydrogel in comparison to C/GB hydrogel (*P* < 0.01) (Figure 2(e)). Thus, we concluded that PF-127 hydrogel exhibited larger porosity and better in vivo biocompatibility, which fulfills the demands for an injectable biomaterial.

### 3.3. Establishment of Thin Endometrium Rat Model

**Figure 3:**
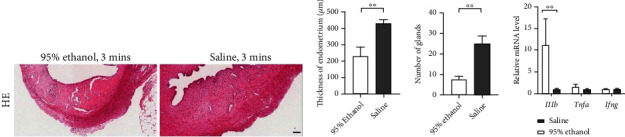
Generation of thin endometrial injury rat model. (a) HE staining of 95% ethanol and saline injection uterine horn. Scale bar = 100 *μ*m. (b) Measurement of endometrium thickness between 95% ethanol and saline injection group. Results were presented as mean ± S.E.M, *n* = 9. Welch's *t*-test was used for significant difference test (∗∗*P* < 0.01). (c) Quantitative analysis of number of glands in each slide between 95% ethanol and saline injection group. Results were presented as mean ± S.E.M, *n* = 9. Student's *t*-test was used for significant difference test (∗∗*P* < 0.01). (d) Q-PCR analyzed the relative gene expression of inflammatory factors (*Il1b*, *Tnfa*, and *Ifng*). Data was reported as mean ± S.E.M. Student's *t*-test was applied for significant difference (∗∗*P* < 0.01 and ns = nonsignificant), *n* = 9.

To characterize the thin endometrium rat model, HE staining and q-PCR were performed on the 95% ethanol and saline-treated uterus. Nine days after the modeling, thinner endometrium thickness was obtained from the ethanol-treated group (232.60 ± 51.62 *μ*m) in comparison to the saline treated group (431.57 ± 22.76 *μ*m) (*P* < 0.01) (Figures 3(a) and 3(b)). The total number of endometrial glands in the ethanol treated group (7.83 ± 1.40) was significantly lower than that in saline treated group (25.33 ± 3.33) (Figure 3(c)). We further performed q-PCR to evaluate inflammatory makers including *Il1b*, *Tnfa*, and *Ifng* in the uterus. Significantly higher expression of *Il1b* was shown in the modeling groups (*P* < 0.01) (Figure 3(d)). Hence, we concluded that we could generate thin endometrium with increased *Il1b* expression level in rat.

### 3.4. Restoration of Endometrium Thickness and Gland Number with Enhanced Vascularization Using PF-127-Encapsulated hUC-MSCs

**Figure 4:**
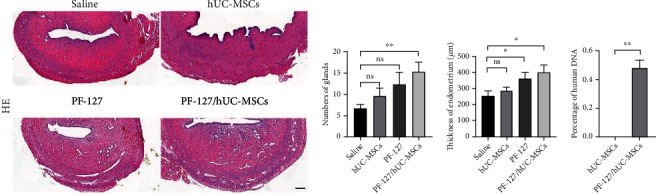
PF-127-encapsulated hUC-MSCs increased endometrial thickness and gland number in the thin endometrium rat model. (a) HE staining of modeling uterine horn with different treatment. Scale bar = 200 *μ*m. (b) Measurement of endometrium thickness from the modeled uterine horn with different treatment. Data was reported as mean ± S.E.M. Student's *t*-test was applied for significant difference (∗*P* < 0.05 and ns = nonsignificant), *n* = 12. (c) Quantitative analysis of number of glands from modeled uterine horn with individual treatment. Data was reported as mean ± S.E.M. Student's *t*-test was applied for significant difference (∗∗*P* < 0.01 and ns = nonsignificant), *n* = 12. (d) Quantitative analysis of human DNA resided in rat uterus. Data was reported as mean ± S.E.M. Mann–Whitney rank sum test was applied for significant difference (∗∗*P* < 0.01), *n* = 12.

To validate whether PF-127-encapsulated hUC-MSCs would help the recovery of thin endometrium, we performed HE staining to examine the morphology of endometrium 9 days after transplantation. Significant increasing of endometrium thickness was observed in PF-127-encapsulated hUC-MSCs transplantation group (401.44 ± 43.57 *μ*m) in comparison to the saline group (251.85 ± 31.17 *μ*m) (*P* < 0.05) (Figures 4(a) and 4(b)). Moreover, the endometrial gland number in PF-127-encapsulated hUC-MSCs transplantation group (15.50 ± 2.15) was significantly higher than that in the saline group (6.75 ± 0.86) (Figure 4(c)). Increasing the trend of gland number was observed in the either hUC-MSCs or PF-127-treated group. Endometrial thickness was able to observe the increasing trend following transplantation with hUC-MSCs alone. However, neither of these two increasing trends achieved a statistically significant difference. Furthermore, we tested the retaining of hUC-MSCs in rat uterus by combining with or without PF-127 using TaqMan qPCR. Nine days after the treatment, only PF-127 and hUC-MSCs cotransplantation group could observe the residing of human DNA (Figure 4(d)). Human DNA was not able to detect in the group with direct hUC-MSCs injection. Thus, our results demonstrated that PF-127 could prolong the retention of hUC-MSCs, which enhanced the endometrium regeneration capacity of hUC-MSCs.

**Figure 5:**
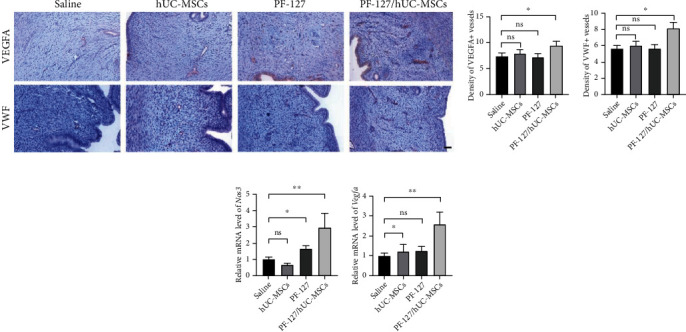
PF-127-encapsulated hUC-MSCs facilitate the angiogenesis of the damaged rat uterine. (a) IHC staining of angiogenesis markers (VWF and VEGFA). Scale bar = 50 *μ*m. (b) Quantitation of VEGFA-positive staining vessels. Data was reported as mean ± S.E.M. Student's *t*-test was applied for significant difference (∗*P* < 0.05 and ns = nonsignificant), *n* = 12. (c) Quantitation of VWF-positive staining vessels. Data was reported as mean ± S.E.M. Student's *t*-test was applied for significant difference (∗*P* < 0.05 and ns = nonsignificant), *n* = 12. (d, e) Q-PCR analyzed the relatively gene expression of angiogenesis markers (*Vegfa* and *Nos3*). The expression values were normalized to 18S. Subsequently, the mRNA expression values were calculated as relative mRNA level versus mRNA expression values with saline injection, which was set to 1. Data was reported as mean ± S.E.M. Student's *t*-test was applied for significant difference (∗∗*P* < 0.01 and ns = nonsignificant), *n* = 12.

Endometrial angiogenesis was one of the critical steps for regeneration. Therefore, IHC and q-PCR were performed to quantify the vascularization of endometrium. Significantly more VEGFA-positive blood vessels were observed in PF-127-encapsulated hUC-MSCs group (9.41 ± 0.77) in comparison to saline-treated group (7.34 ± 0.62) (*P* < 0.05) (Figures 5(a) and 5(b)). The same trend was also observed in VWF-positive blood vessels, in which strong VWF-positive blood vessels were found in hydrogel embedded hUC-MSCs group (8.143 ± 0.79) (Figures 5(a) and 5(c)). To further confirm the vascularization capacity, gene expressions of angiogenesis markers *Vegfa* and *Nos3* were evaluated with q-PCR. Similarly, gene expressions of *Vegfa* and *Nos3* were significantly increased in PF-127-encapsulated hUC-MSCs group compared to saline group (*P* < 0.05) (Figures 5(d) and 5(e)). These data indicated that hUC-MSCs embedded in thermosensitive hydrogel could regenerate thin endometrium with enhanced angiogenesis.

### 3.5. IL-1*β*-Primed hUC-MSCs Improve Angiogenesis via Releasing of Neovascularization Factors

**Figure 6:**
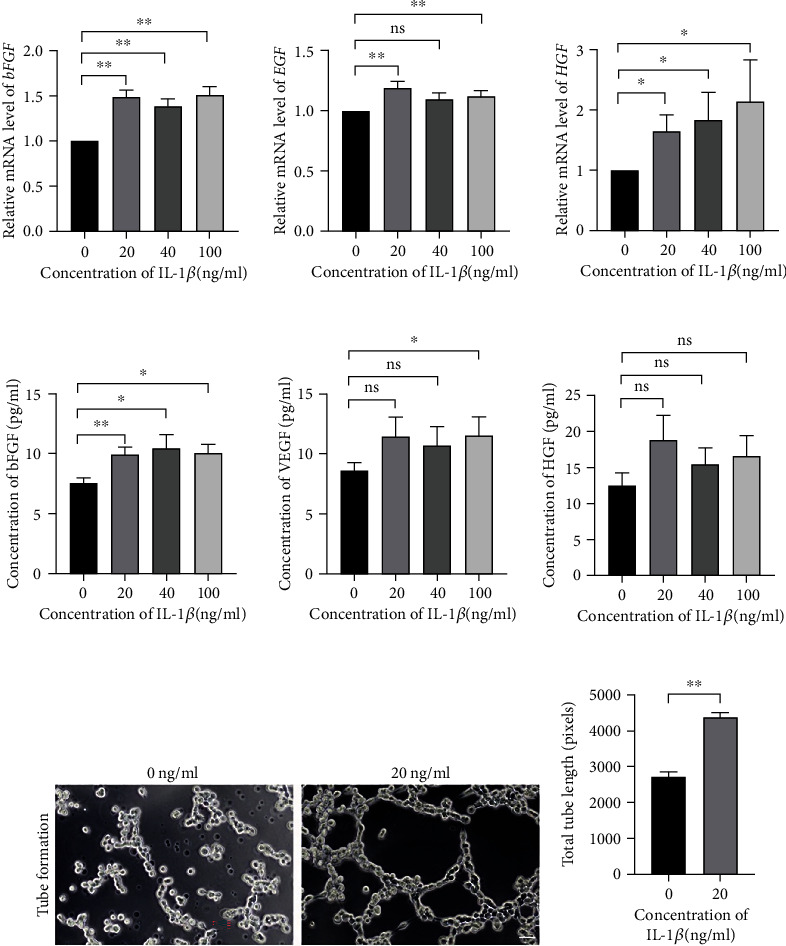
HUC-MSCs responded to the IL-1*β* microenvironment with the releasing of neovascularization factors to promote angiogenesis. (a) Quantitation of the relative gene expression of angiogenesis markers (*bFGF*, *EGF*, and *HGF*) in the hUC-MSCs with the various concentration of IL-1*β* induction. Data was reported as mean ± S.E.M of 3 independent cultures, tested for significant difference (∗*P* < 0.05, ∗∗*P* < 0.01, ns = nonsignificant) using Student's *t*-test. (b) Luminex analyzed the protein concentration of angiogenesis markers (bFGF, VEGF, and HGF) in the stimulated hUC-MSCs supernatant. Data was reported as mean ± S.E.M of 3 independent cultures supernatant, tested for significant difference (∗*P* < 0.05, ∗∗*P* < 0.01, ns = nonsignificant) using Student's *t*-test. (c) Phase contrast images of HUVEC cultured in the hUC-MSCs supernatant with or without the IL-1*β* induction. Scale bar = 50 *μ*m. (d) Quantification of tube length of HUVEC culturing within the supernatant from the hUC-MSCs with or without the IL-1*β* induction. Results were presented as mean ± S.E.M of approximately 60 pictures from 3 independent cultures, tested for significant difference (∗∗*P* < 0.01) using Mann–Whitney rank sum test.

Local microenvironmental cues could induce MSCs to release bioactive factors and signals to regenerate the damaged tissues. Our data indicated that high *Il1b* expression was observed in modeled uterus. Meanwhile, enhanced angiogenesis was found in PF-127-encapsulated hUC-MSCs group. Variable concentrations of IL-1*β* (0, 20, 40, and 100 ng/ml) were applied to prime hUC-MSCs for mimicking the hUC-MSCs in response to the local environment of modeled uterus. To investigate the angiogenesis role of IL-1*β*-primed hUC-MSCs, q-PCR was carried out to evaluate the expression of angiogenesis genes (*bFGF*, *EGF*, and *HGF*). Significantly upregulated *bFGF* was found in the hUC-MSCs treated with different concentrations of IL-1*β* (*P* < 0.01) (Figure 6(a)). *EGF* was increased significantly in hUC-MSCs incubation with 20 and 100 ng/ml of IL-1*β* (*P* < 0.01) (Figure 6(a)). Expression level of *HGF* was significantly upregulated in the hUC-MSCs primed with IL-1*β* (*P* < 0.05) (Figure 6(a)). To further confirm the paracrine functions of hUC-MSCs stimulated with IL-1*β*, Luminex was performed to evaluate the concentration of angiogenesis protein (bFGF, VEGF, and HGF). Luminex data showed significantly increased bFGF concentration in the supernatant with IL-1*β* stimulation in comparison to that without stimulation (IL − 1*β* = 20 ng/ml, *P* < 0.01; IL − 1*β* = 40 ng/ml and 100 ng/ml, *P* < 0.05) (Figure 6(b)). Significantly increased concentration of VEGF was observed in the medium derived from 100 ng/ml IL-1*β*-primed hUC-MSCs (*P* < 0.05) (Figure 6(b)). Increased concentration of HGF was found in the medium originated from IL-1*β*-stimulated hUC-MSCs (Figure 6(b)). Moreover, supernatant from 20 ng/ml IL-1*β*-primed hUC-MSCs was used to measure the angiogenesis potential with tube formation assays in vitro. Total tube length was significantly increased in HUVEC incubated in the medium derived from 20 ng/ml IL-1*β*-primed hUC-MSCs (*P* < 0.01) (Figures 6(c) and 6(d)). Our data indicated that hUC-MSCs in response to local highly expressed IL-1*β* promoted neovascularization potential.

## 4. Discussion

Stem cell therapy has been used in restoring the function of damaged tissues [10]. However, limited MSCs could arrive or survive in the uterus by intravenous administration or local infusion [11]. Previous study indicated that biocompatible scaffolds encapsulated MSCs could significantly improve the treatment efficiency via prolonging cell retention and enhancing cell survival in vivo [38]. Therefore, we constructed thermosensitive hydrogel and hUC-MSCs for regeneration of thin endometrium modeled rat uterus. Our results clearly demonstrated that thermosensitive PF-127-encapsulated hUC-MSCs had long in vivo residing time, which could further alleviate thin endometrium with increased endometrium thickness, endometrial gland number, and vascularization capacity. Moreover, IL-1*β* microenvironment could induce hUC-MSCs to release angiogenesis factors, which played a key role during thin endometrium recovery.

MSCs could be isolated from various tissues, such as bone marrow, adipose tissue, periapical cyst, dental pulp, placenta, and umbilical cord. However, the MSCs derived from different tissues would exhibit varying therapeutic effects with different diseases. Human periapical cyst-derived MSCs (hPCy-MSCs) could differentiate into dopaminergic neurons, which play an important role in brain regeneration and neurodegenerative disease modeling [39, 40]. MSCs isolated from umbilical cord are able to differentiate into endometrial epithelial cell (EEC)-like and endometrial stromal cell (ESC)-like cells in vitro [41]. Previous study indicated that MSCs derived from various tissues were characterized with positive CD105, CD90, and CD73 and negative hematopoietic cells including HLA-DR, CD45, and CD34 [42]. In accordance with previous study, our data demonstrated that the MSCs isolated from umbilical cord expressed the typical MSCs surface makers and multilineage differentiation. In this light, hUC-MSCs characterized with extensive multipotency and high proliferative capacity would make it the most useful stem cell type in regeneration medicine in thin endometrium.

Thermosensitive hydrogels, characterized as liquid phase at low temperature and solid phase when temperature increasing, could develop equivalent hydrogel to cover the whole uterus [43]. Thermosensitive PF-127 and C/GP hydrogel were used to embed MSCs for tissues regeneration. Previous study indicated that constructing PF-127 with adipose-derived stem cell (ADSCs) was able to promote wound healing and cell proliferation [44]. PF-127 has been employed for in vitro differentiation scaffold for bone marrow-derived MSCs and dental-derived MSCs [45, 46]. C/GP thermosensitive hydrogel was applied to encapsulate MSCs for in vitro proliferation and osteogenic differentiation [47, 48]. Our finding showed that PF-127 takes shorter time to form solid hydrogel compared to C/GB hydrogel. These characters ensured that PF-127 could develop into solid hydrogel in the uterus within a short period of time without leaking from the uterus during clinic application in contrast to C/GB. In our study, SEM results demonstrated that 3D porous scaffold could be observed in both PF-127 and C/GB hydrogel. Three-dimensional porous structure scaffold promoted proliferation and differentiation of MSCs [34]. Our data indicated that significantly higher porosity was found in PF-127 compared to C/GB hydrogel. Larger porosity would be beneficial for biofactors releasing [34]. Four weeks after injection of PF-127 and C/GB hydrogel, significantly higher expression of inflammatory markers (*Tnfa*, *Ifng*, and *Il2*) was observed in the surrounding tissues with C/GB hydrogel injection in comparison to PF-127 injection. Our data indicated that high porosity and biocompatible PF-127 would be more suitable for encapsulation of hUC-MSCs for thin endometrium regeneration.

Thin endometrium was associated with lower clinical pregnancy and birth weight during the IVF-ET cycles [3, 49, 50]. Similar to previous studies [51, 52], we modeled the thin endometrium by infusing the endometrium with 95% ethanol resulting in reduced endometrium thickness and gland number. Similar to the thin endometrium uterus environment, significantly higher expression of inflammatory marker *Il1b* was observed in the uterus after thin endometrium modeling [53, 54]. Previous study indicated that increased endometrium thickness could be observed after 12-day uterine injection [55]. Similar to previous study, our data could observe the increasing trend of endometrium thickness, gland number, and newly generated blood vessels after 9 days of hUC-MSCs injection. However, this increasing trend was not significant. Our TaqMan qPCR results indicated that human-specific DNA was not able to detect after 9 days of only hUC-MSCs transplantation group. Cotransplanted hUC-MSCs with PF-127 could retain in uterus for 9 days. Thus, the therapeutics effective of only hUC-MSCs injection would be very limited. Significantly increased endometrial thickness and gland number was only achieved by transplantation of PF-127/hUC-MSCs.

Thin endometrium was highly characterized by poor vascular development, decreased VEGF expression, and poor epithelial growth [56]. Therefore, neovascularization is one of the critical steps for endometrium regeneration. Combination of scaffolds and MSCs could induce neovascularization potential of scared uterus via increasing expression of new blood vessel makers (VWF) [20, 57]. Consistent with previous study, significantly increased vascular markers abundancy (*Vegfa* and *Nos3*) was observed in the thin endometrium modeled uterus with PF-127/hUC-MSCs treatment. Moreover, significantly more VWF- and VEGFA-positive blood vessels were confirmed by IHC staining after the modeled thin endometrium were treated with PF-127/hUC-MSCs. Thus, our results indicated that PF-127 encapsulation of hUC-MSCs could regenerate thin endometrium via promoting angiogenesis.

Reduced blood flow induced hypoxia in thin endometrium patient uterus [58]. Genome-wide single-cell RNA sequencing revealed decreased macrophages and natural killer cells in the human thin endometrium [59]. Highly expressed inflammatory genes including *TNF-α*, *IFN-γ,* and *interleukin 1 receptor* (*IL1R1*) were observed in thin endometrium through genomic mRNA sequencing analysis [53, 54]. Previous study also indicated that local inflammatory environment was associated with occurrence of metabolic acidosis and shifting of extracellular pH [60]. MSCs could be stimulated by local injury microenvironment to release growth factors, exosomes, and complements to execute the therapeutic effects during regeneration [61]. Acidosis-primed MSCs could release the specified exosomes, which could increase the anti-inflammatory T cell subtypes in vitro [62]. Enhanced proliferation and neovascularization capacity were reported in the MSCs primed by hypoxia [63]. Inflammatory cytokine IL-1*β*-primed hUC-MSCs could release of growth factors to promote epidermal substitute engraftment and wound healing in vivo [64]. Significantly increased expression of *Il1b* in the modeled rat uterus might be the local stimulation molecules to promote hUC-MSCs therapeutic role. Our data indicated that upregulated expression levels of angiogenetic markers *bFGF*, *EGF*, and *HGF* were observed in the hUC-MSCs stimulated with IL-1*β*, which is in accordance with previous studies [65, 66]. Moreover, enhanced secretion of bFGF, EGF, and VEGF was detected in medium derived from the IL-1*β*-primed hUC-MSCs, as previously reported [64]. Collagen-binding bFGF could regenerate the damaged uterus by promoting angiogenesis with increased blood vessels [67]. Intravenously administrated VEGF overexpressed MSCs could increase the endometrium thickness [52]. The expressions of EGF and HGF were required for the proliferation of endometrium of epithelial and stromal cells [68, 69]. VEGF exhibited a vital role in endometrium regeneration, which was found highly expressed in human endometrium [70, 71]. Since these angiogenic factors could promote proliferation, migration, and composition of capillary tubes, we further compared the neovascularization potential of medium derived from IL-1*β*-primed hUC-MSCs and nonprimed hUC-MSCs. Our results clearly proved that enhanced angiogenesis capacity was found in IL-1*β*-primed hUC-MSCs medium in tube formation assay. Taken together, our results demonstrated that the hUC-MSCs could respond to local IL-1*β* microenvironment, which promoted the release of angiogenic factors to enhance thin endometrium regeneration.

There are some limitations that need to be addressed in our study. Our data suggested that IL-1*β* environment could promote hUC-MSCs to secrete the angiogenesis cytokines to regenerate thin endometrium. The underlying molecular mechanisms of thin endometrium regeneration need to be further elucidated. Overexpressions of upregulated molecules including bFGF, EGF, HGF, and VEGF in hUC-MSCs should be tested in thin endometrium regeneration. Furthermore, to generalize environment stimulation on therapeutic role in MSCs, the mass spectrometric and transcriptome sequencing could be performed to compare the difference of secretome of hUC-MSCs with or without IL-1*β* stimulation. Moreover, the immune modulation and angiogenesis potential of stem cell would elevate the tumorigenesis risk during stem cell therapy. Previous studies indicated that local microenvironment could stimulate MSCs to secrete exosomes, which play a key role in stem cell therapy [22]. Therefore, in future studies, we plan to clarify that if application of exosomes released from IL-1*β*-primed hUC-MSCs on regeneration of thin endometrium to improve the safety and efficacy of our stem cell therapy.

Human umbilical cord tissue derived from different donors would produce hUC-MSCs with functional heterogeneity, which could bring therapeutic variations [72]. HUC-MSCs derived from different donors should be tested for their thin endometrium regeneration potential to make sure the therapeutic consistency. Moreover, hUC-MSCs have to be manufactured under GMP conditions for clinical application.

## 5. Conclusions

In this study, we found that PF-127 encapsulation of hUC-MSCs could restore the morphology of modeled thin endometrium, with increased endometrium thickness and gland number, and promoted neovascularization capacity. We further uncovered that local IL-1*β* microenvironment stimulated the hUC-MSCs to release angiogenic and endometrium regeneration factors to regenerate the structure of thin endometrium. Overall, we identify a novel underlying mechanism of combination of hUC-MSCs and biomaterials to regenerate thin endometrium.

## Figures and Tables

**Table 1:** Primer sequences.

Primer name	Primer sequence	Gene name
Rat-*Tnfa*-F	CCCAATCTGTGTCCTTCTAACT	Tumor necrosis factor-alpha
Rat-*Tnfa*-R	CAGCGTCTCGTGTGTTTCT	
Rat-*Ifng*-F	GTGAACAACCCACAGATCCA	Interferon gamma
Rat-*Ifng*-R	GAATCAGCACCGACTCCTTT	
Rat-*Il2*-F	GCAGGCCACAGAATTGAAAC	Interleukin-2
Rat-*Il2*-R	CCAGCGTCTTCCAAGTGAA	
Rat-*Il1b*-F	CGACAGTGAGGAGAATGACC	Interleukin-1 beta
Rat-*Il1b*-R	CACAGCCACAATGAGTGACAC	
Rat-*Nos3*-F	TGGGAGGGAGTCAGCCTAAA	Nitric oxide synthase 3
Rat-*Nos3*-R	AAGTGTTGGGTTGGGCATCTC	
Rat-*Vegfa*-F	GAAGACACAGTGGTGGAAGAAG	Vascular endothelial growth factor A
Rat-*Vegfa*-R	ACAAGGTCCTCCTGAGCTATAC	
Rat-*18S*-F	CGGCTACCACATCCAAGGA	RNA, 18S ribosomal N1
Rat-*18S*-R	CCAATTACAGGGCCTCGAAA	
Human-*bFGF*-F	AGAAGAGCGACCCTCACATCA	Fibroblast growth factor 2
Human-*bFGF*-R	CGGTTAGCACACACTCCTTTG	
Human-*EGF*-F	TCCTCACCCGATAATGGTGGA	Epidermal growth factor
Human-*EGF*-R	CCAGGAAAGCAATCACATTCCC	
Human-*HGF*-F	GCTATCGGGGTAAAGACCTACA	Hepatocyte growth factor
Human-*HGF*-R	CGTAGCGTACCTCTGGATTGC	
Human-*B2M* -F	GATGAGTATGCCTGCCGTGT	Beta-2 microglobulin
Human-*B2M*-R	TGCGGCATCTTCAAACCTCC	

**Table 2:** Oligonucleotides and probes used for TaqMan qPCR.

Name	5′ to 3′ sequence
a-satellite F	CTCTCTTTCTGTGGCATCTGCA
a-satellite R	ACGAAGGACACAGAGTGGTCC
a-satellite probe	FAM-CACGTTTGAAACACTC-MGB-NFQ

## Data Availability

The primary data presented in this study are available on request from the corresponding author.

## References

[1] Yuan X., Saravelos S. H., Wang Q., Xu Y., Li T. C., Zhou C. (2016). Endometrial thickness as a predictor of pregnancy outcomes in 10787 fresh IVF- ICSI cycles. *reproductive biomedicine online*.

[2] Liu H., Zhang J., Wang B., Kuang Y. (2020). Effect of endometrial thickness on ectopic pregnancy in frozen embryo transfer cycles: an analysis including 17,244 pregnancy cycles. *fertility and sterility*.

[3] Liu K. E., Hartman M., Hartman A., Luo Z. . C., Mahutte N. (2018). The impact of a thin endometrial lining on fresh and frozen-thaw IVF outcomes: an analysis of over 40 000 embryo transfers. *Human Reproduction*.

[4] Kasius A., Smit J. G., Torrance H. L. (2014). Endometrial thickness and pregnancy rates after IVF: a systematic review and meta-analysis. *Human Reproduction Update*.

[5] Ribeiro V. C., Santos-Ribeiro S., de Munck N. (2018). Should we continue to measure endometrial thickness in modern-day medicine? The effect on live birth rates and birth weight. *Reproductive Biomedicine Online*.

[6] Groothuis P. G., Dassen H. H. N. M., Romano A., Punyadeera C. (2007). Estrogen and the endometrium: lessons learned from gene expression profiling in rodents and human. *Human Reproduction Update*.

[7] Ranisavljevic N., Raad J., Anahory T., Grynberg M., Sonigo C. (2019). Embryo transfer strategy and therapeutic options in infertile patients with thin endometrium: a systematic review. *Journal of Assisted Reproduction and Genetics*.

[8] Andrzejewska A., Lukomska B., Janowski M. (2019). Concise review: mesenchymal stem cells: from roots to boost. *Stem Cells*.

[9] Hendijani F. (2017). Explant culture: an advantageous method for isolation of mesenchymal stem cells from human tissues. *Cell Proliferation*.

[10] Naji A., Eitoku M., Favier B., Deschaseaux F., Rouas-Freiss N., Suganuma N. (2019). Biological functions of mesenchymal stem cells and clinical implications. *Cellular and Molecular Life Sciences*.

[11] Schmuck E. G., Koch J. M., Centanni J. M. (2016). Biodistribution and clearance of human mesenchymal stem cells by quantitative three-dimensional cryo-imaging after intravenous infusion in a rat lung injury model. *Stem Cells Translational Medicine*.

[12] Hong K. H., Kim Y. M., Song S. C. (2019). Fine-tunable and injectable 3D hydrogel for on-demand stem cell niche. *Adv Sci (Weinh)*.

[13] Adams J. R., Senapati S., Haughney S. L., Wannemuehler M. J., Narasimhan B., Mallapragada S. K. (2019). Safety and biocompatibility of injectable vaccine adjuvants composed of thermogelling block copolymer gels. *Journal of Biomedical Materials Research. Part A*.

[14] Derakhshandeh K., Fashi M., Seifoleslami S. (2010). Thermosensitive Pluronic hydrogel: prolonged injectable formulation for drug abuse. *Drug Design, Development and Therapy*.

[15] Akash M. S., Rehman K., Sun H., Chen S. (2013). Sustained delivery of IL-1Ra from PF127-gel reduces hyperglycemia in diabetic GK-rats. *PLoS One*.

[16] Yang J., Chen Z., Pan D., Li H., Shen J. (2020). Umbilical cord-derived mesenchymal stem cell-derived exosomes combined Pluronic F127 hydrogel promote chronic diabetic wound healing and complete skin regeneration. *International Journal of Nanomedicine*.

[17] Silva A. K. A., Perretta S., Perrod G. (2018). Thermoresponsive gel embedded with adipose stem-cell-derived extracellular vesicles promotes esophageal fistula healing in a thermo-actuated delivery strategy. *ACS Nano*.

[18] Brunet-Maheu J. M., Fernandes J. C., de Lacerda C. A. V., Shi Q., Benderdour M., Lavigne P. (2009). Pluronic F-127 as a cell carrier for bone tissue engineering. *Journal of Biomaterials Applications*.

[19] Chen W. J., Huang J. W., Niu C. C. (2009). Use of fluorescence labeled mesenchymal stem cells in Pluronic F127 and porous hydroxyapatite as a bone substitute for posterolateral spinal fusion. *Journal of Orthopaedic Research*.

[20] Yang H., Wu S., Feng R. (2017). Vitamin C plus hydrogel facilitates bone marrow stromal cell-mediated endometrium regeneration in rats. *Stem Cell Research & Therapy*.

[21] Fan X.-L., Zhang Y., Li X., Fu Q. L. (2020). Mechanisms underlying the protective effects of mesenchymal stem cell-based therapy. *Cellular and molecular life sciences : CMLS*.

[22] Kusuma G. D., Carthew J., Lim R., Frith J. E. (2017). Effect of the microenvironment on mesenchymal stem cell paracrine signaling: opportunities to engineer the therapeutic effect. *Stem Cells and Development*.

[23] Bressan E., Ferroni L., Gardin C. (2019). Metal nanoparticles released from dental implant surfaces: potential contribution to chronic inflammation and peri-implant bone loss. *Materials (Basel)*.

[24] Kwon Y. W., Heo S. C., Jeong G. O. (2013). Tumor necrosis factor-*α*-activated mesenchymal stem cells promote endothelial progenitor cell homing and angiogenesis. *Biochimica et Biophysica Acta (BBA) - Molecular Basis of Disease*.

[25] Kanai R., Nakashima A., Doi S. (2021). Interferon-*γ* enhances the therapeutic effect of mesenchymal stem cells on experimental renal fibrosis. *Scientific Reports*.

[26] Council, NR (2010). *Guide for the care and use of laboratory animals*.

[27] Karlsson C., Emanuelsson K., Wessberg F. (2009). Human embryonic stem cell-derived mesenchymal progenitors—potential in regenerative medicine. *Stem cell research*.

[28] Kang R., Zhou Y., Tan S. (2015). Mesenchymal stem cells derived from human induced pluripotent stem cells retain adequate osteogenicity and chondrogenicity but less adipogenicit. *Stem Cell Research & Therapy*.

[29] Zhang K., Zhao X., Chen X. (2018). Enhanced therapeutic effects of mesenchymal stem cell-derived exosomes with an injectable hydrogel for hindlimb ischemia treatment. *ACS applied materials & interfaces*.

[30] Song K., Qiao M., Liu T. (2010). Preparation, fabrication and biocompatibility of novel injectable temperature-sensitive chitosan/glycerophosphate/collagen hydrogels. *Journal of Materials Science: Materials in Medicine*.

[31] Song K., Li, Yan X. (2017). Characterization of human adipose tissue-derived stem cells in vitro culture and in vivo differentiation in a temperature-sensitive chitosan/*β*\- glycerophosphate/collagen hybrid hydrogel. *Materials Science and Engineering: C*.

[32] Schindelin J., Arganda-Carreras I., Frise E. (2012). Fiji: an open-source platform for biological-image analysis. *Nature methods*.

[33] Alemdar N., Leijten J., Camci-Unal G. (2017). Oxygen-generating photo-cross-linkable hydrogels support cardiac progenitor cell survival by reducing hypoxia-induced necrosis. *ACS Biomaterials Science & Engineering*.

[34] Loh Q. L., Choong C. (2013). Three-dimensional scaffolds for tissue engineering applications: role of porosity and pore size. *Tissue Engineering. Part B, Reviews*.

[35] Livak K. J., Schmittgen T. D. (2001). Analysis of Relative Gene Expression Data Using Real-Time Quantitative PCR and the 2^−*ΔΔC*^_T_ Method. *methods*.

[36] Warburton P. E., Greig G. M., Haaf T., Willard H. F. (1991). PCR amplification of chromosome-specific alpha satellite DNA: definition of centromeric STS markers and polymorphic analysis. *Genomics*.

[37] Kleinman H. (2010). In vitro angiogenesis: endothelial cell tube formation on gelled basement membrane extract. *Nature Protocols*.

[38] Ma T., Wu J., Mu J., Gao J. (2022). Biomaterials reinforced MSCs transplantation for spinal cord injury repair. *Asian Journal of Pharmaceutical Sciences*.

[39] Tatullo M., Codispoti B., Pacifici A. (2017). Potential use of human periapical cyst-mesenchymal stem cells (hPCy-MSCs) as a novel stem cell source for regenerative medicine applications. *Frontiers in Cell and Developmental Biology*.

[40] Tatullo M., Marrelli B., Zullo M. J. (2020). Exosomes from human periapical cyst-MSCs: theranostic application in Parkinson’s disease. *International Journal of Medical Sciences*.

[41] Shi Q., Gao J. W., Jiang Y. (2017). Differentiation of human umbilical cord Wharton’s jelly-derived mesenchymal stem cells into endometrial cells. *Stem Cell Research & Therapy*.

[42] Barry F. P. (2003). Biology and clinical applications of mesenchymal stem cells. *Birth Defects Research. Part C, Embryo Today*.

[43] Li D. (2008). *Thermosensitive Hydrogels, in Encyclopedia of Microfluidics and Nanofluidics*.

[44] Kaisang L., Siyu W., Lijun F., Daoyan P., Xian C. J., Jie S. (2017). Adipose-derived stem cells seeded in Pluronic F-127 hydrogel promotes diabetic wound healing. *Journal of surgical research*.

[45] Vashi A. V., Keramidaris E., Abberton K. M. (2008). Adipose differentiation of bone marrow-derived mesenchymal stem cells using Pluronic F-127 hydrogel *in vitro*. *biomaterials*.

[46] Diniz I. M. A., Chen C., Xu X. (2015). Pluronic F-127 hydrogel as a promising scaffold for encapsulation of dental-derived mesenchymal stem cells. *Journal of materials science materials in medicine*.

[47] Yang M., He S., Su Z., Yang Z., Liang X., Wu Y. (2020). Thermosensitive injectable chitosan/collagen/*β*-glycerophosphate composite hydrogels for enhancing wound healing by encapsulating mesenchymal stem cell spheroids. *ACS Omega*.

[48] Saravanan S., Vimalraj S., Thanikaivelan P., Banudevi S., Manivasagam G. (2019). A review on injectable chitosan/beta glycerophosphate hydrogels for bone tissue regeneration. *International journal of biological macromolecules*.

[49] Zhang J., Liu H., Mao X. (2019). Effect of endometrial thickness on birthweight in frozen embryo transfer cycles: an analysis including 6181 singleton newborns. *Human Reproduction*.

[50] Guo Z., Xu X., Zhang L., Zhang L., Yan L., Ma J. (2020). Endometrial thickness is associated with incidence of small-for-gestational- age infants in fresh in vitro fertilization-intracytoplasmic sperm injection and embryo transfer cycles. *Fertility and Sterility*.

[51] Xia L., Meng Q., Xi J. (2019). The synergistic effect of electroacupuncture and bone mesenchymal stem cell transplantation on repairing thin endometrial injury in rats. *Stem Cell Research & Therapy*.

[52] Jing Z., Yi Y., Xi H., Sun L. Q., Yanping L. (2018). Therapeutic effects of VEGF gene-transfected BMSCs transplantation on thin endometrium in the rat model. *Stem Cells International*.

[53] Maekawa R., Taketani T., Mihara Y. (2017). Thin endometrium transcriptome analysis reveals a potential mechanism of implantation failure. *Reproductive Medicine and Biology*.

[54] Zong L., Zheng S., Meng Y. (2021). Integrated transcriptomic analysis of the miRNA-mRNA interaction network in thin endometrium. *Frontiers in Genetics*.

[55] Zhao J., Zhang Q., Wang Y., Li Y. (2015). Uterine infusion with bone marrow mesenchymal stem cells improves endometrium thickness in a rat model of thin endometrium. *Reproductive sciences (Thousand Oaks, Calif.)*.

[56] Miwa I., Tamura H., Takasaki A., Yamagata Y., Shimamura K., Sugino N. (2009). Pathophysiologic features of "thin" endometrium. *Fertility and Sterility*.

[57] Xu L., Ding L., Wang L. (2017). Umbilical cord-derived mesenchymal stem cells on scaffolds facilitate collagen degradation via upregulation of MMP-9 in rat uterine scars. *stem cell research & therapy*.

[58] Hickey M., Krikun G., Kodaman P., Schatz F., Carati C., Lockwood C. J. (2006). Long-term progestin-only contraceptives result in reduced endometrial blood flow and oxidative stress. *The Journal of Clinical Endocrinology and Metabolism*.

[59] Lv H., Zhao G., Jiang P. (2022). Deciphering the endometrial niche of human thin endometrium at single-cell resolution. *Proceedings of the National Academy of Sciences*.

[60] Erra Díaz F., Dantas E., Geffner J. (2018). Unravelling the interplay between extracellular acidosis and immune cells. *Mediators of Inflammation*.

[61] Shi Y., Wang Y., Li Q. (2018). Immunoregulatory mechanisms of mesenchymal stem and stromal cells in inflammatory diseases. *Nature Reviews Nephrology*.

[62] Andrews S., Maughon T., Marklein R., Stice S. (2021). Priming of MSCs with inflammation-relevant signals affects extracellular vesicle biogenesis, surface markers, and modulation of T cell subsets. *Journal of Immunology and Regenerative Medicine*.

[63] Nowak-Stępniowska A., Osuchowska P. N., Fiedorowicz H., Trafny E. A. (2022). Insight in hypoxia-mimetic agents as potential tools for mesenchymal stem cell priming in regenerative medicine. *Stem Cells International*.

[64] Magne B., Dedier M., Nivet M. (2020). IL-1*β*-primed mesenchymal stromal cells improve epidermal substitute engraftment and wound healing via matrix metalloproteinases and transforming growth factor-*β*1. *Journal of Investigative Dermatology*.

[65] Broekman W., Amatngalim G. D., de Mooij-Eijk Y. (2016). TNF-*α* and IL-1*β*-activated human mesenchymal stromal cells increase airway epithelial wound healing in vitro via activation of the epidermal growth factor receptor. *Respiratory Research*.

[66] Ghandadi M., Sahebkar A. (2017). Curcumin: an effective inhibitor of interleukin-6. *Current Pharmaceutical Design*.

[67] Li X., Sun H., Lin N. (2011). Regeneration of uterine horns in rats by collagen scaffolds loaded with collagen-binding human basic fibroblast growth factor. *Biomaterials*.

[68] Gargett C. E., Chan R. W., Schwab K. E. (2008). Hormone and growth factor signaling in endometrial renewal: role of stem/progenitor cells. *Molecular and Cellular Endocrinology*.

[69] Islam M. R., Yamagami K., Yoshii Y., Yamauchi N. (2016). Growth factor induced proliferation, migration, and lumen formation of rat endometrial epithelial cells in vitro. *The Journal of Reproduction and Development*.

[70] Lash G. E., Innes B. A., Drury J. A., Robson S. C., Quenby S., Bulmer J. N. (2012). Localization of angiogenic growth factors and their receptors in the human endometrium throughout the menstrual cycle and in recurrent miscarriage. *Human Reproduction*.

[71] Fan X., Krieg S., Kuo C. J. (2008). VEGF blockade inhibits angiogenesis and reepithelialization of endometrium. *The FASEB Journal*.

[72] Xie Y., Liu S., Wang L. (2021). Individual heterogeneity screened umbilical cord-derived mesenchymal stromal cells with high Treg promotion demonstrate improved recovery of mouse liver fibrosis. *Stem Cell Research & Therapy*.

